# Adapting the log quadratic model to estimate age- and cause-specific mortality among neonates

**DOI:** 10.1371/journal.pone.0304841

**Published:** 2024-07-12

**Authors:** Jamie Perin, Li Liu, Luke C. Mullany, James M. Tielsch, Andrea Verhulst, Michel Guillot, Joanne Katz

**Affiliations:** 1 Department of International Health, Johns Hopkins Bloomberg School of Public Health, Baltimore, Maryland, United States of America; 2 Department of Population, Family and Reproductive Health, Johns Hopkins Bloomberg School of Public Health, Baltimore, Maryland, United States of America; 3 Department of Global Health, Milken Institute School of Public Health, George Washington University, Washington, DC, United States of America; 4 Institut National d’Études Démographiques, Aubervilliers, France; 5 Population Studies Center, University of Pennsylvania, Philadelphia, Pennsylvania, United States of America; King Faisal University, SAUDI ARABIA

## Abstract

**Introduction:**

Estimates for cause-specific mortality for neonates are generally available for all countries for neonates overall (0 to 28 days). However, cause-specific mortality is generally not being estimated at higher age resolution for neonates, despite evidence of heterogeneity in the causes of deaths during this period. We aimed to use the adapted log quadratic model in a setting where verbal autopsy was the primary means of determining cause of death.

**Methods:**

We examined the timing and causes of death among a cohort of neonates in rural Nepal followed as part of the Nepal Oil Massage Study (NOMS). We adapted methods defined by Wilmoth et al (2012) and Guillot et al. (2022) to estimate age and cause-specific mortality among neonates. We used cross validation to estimate the accuracy of this model, holding out each three month period. We took the average cross validation across hold out as our measure of model performance and compared to a standard approach which did not account for the heterogeneity in cause-specific mortality rate within this age group.

**Results:**

There were 957 neonates in the NOMS cohort with known age and cause of death. We estimated an average cross-validation error of 0.9 per 1000 live births for mortality due to prematurity in the first week, and 1.1 for mortality due to birth asphyxia, compared to the standard approach, having error 7.4 and 7.8 per 1000 live births, respectively. Generally mortality rates for less common causes such as congenital malformations and pneumonia were estimated with higher cross-validation error.

**Conclusions:**

The stability and precision of these estimates compare favorably with similar estimates developed with higher quality cause-specific mortality surveillance from China, demonstrating that reliably estimating causes of mortality at high resolution is possible for neonates in low resources areas.

## Introduction

Cause-specific mortality rates for children under five at the national level for all countries are currently available both for neonates (in the first month or 28 days of life) and also for children aged one to 59 months (the remainder of under-five deaths) [1]. These estimates are made publicly available to policy makers, health planners, and the international health community so that the most appropriate interventions can be identified and targeted where the most lives can be saved [2]. The Sustainable Development Goals (SDG) have specified targets for under-five and neonatal mortality at 25 and 12 deaths per 1000 live births as part of SDG 3.1.2 [3]. Progress toward the SDG goals critically depends on the availability of these estimates and their applicability to the ground truth for both neonates and children under-five.

Neonatal mortality in particular is becoming more predominant among under-five mortality, as mortality for older children, often attributable to infectious conditions such as acute respiratory illness, diarrhea, and malaria, is in steep decline in many areas [1, 4]. If recent progress in the decline of under-five mortality is to continue, effective interventions for neonates will be needed, and interventions will need to be directed to the areas and populations that need them most. If cause- and age-specific mortality rate estimates were available for neonates at higher resolution by age (for example, by day, or by week) within the first month of life, this information could be used in health system and program planning to save more lives. For example, if deaths due to sepsis occur primarily in the first day of life, promotion of facility delivery would be the primary means of intervention to reduce sepsis-related mortality, as the health system may otherwise be unlikely to have sufficient time for contact. In contrast, if mortality due to sepsis occurred primarily later in the neonatal period, postnatal home or facility visits would be an important addition to facility delivery. In addition, the actual ages for neonatal mortality due to sepsis may be variable over time or across countries, such that the best strategy to address sepsis-related neonatal mortality would be specific to setting.

Recently, Guillot et al (2022) [5], building on an earlier study by Wilmoth (2012) [6], developed a log-quadratic model to estimate all-cause mortality by detailed age between ages 0 and 5 years. In prior work, we adapted Guillot et al.’s log-quadratic model to estimate cause-specific mortality for children under five for cause-specific mortality [7]. However, this work for cause-specific under-five mortality only examined two age groups within neonates, and focused on causes that were most common among all under-five children. In addition, this research utilized mortality data from the Maternal and Child Health Surveillance System (MCHSS) in China, which uses medically registered causes of death for approximately 80% of under-five deaths [8]. In addition, for these methods to be truly useful for reducing neonatal mortality in countries with a high neonatal mortality rate, we need to know that they can be applied to lower quality mortality data such as that using verbal autopsies for determining the causes of neonatal mortality at high age resolution.

We examined age- and cause-specific mortality in a cohort in Nepal where neonatal mortality was high, high quality mortality surveillance identified a population representative set of neonatal deaths, and causes of death were estimated with verbal autopsies. The cause of death distribution overall among this group of neonates has been previously reported [9]. We aimed to estimate the log-quadratic model for high resolution age groups for cause-specific neonatal mortality in this high mortality setting. In addition, we aimed to determine the validity of the log-quadratic model more generally for age- and cause-specific mortality as adapted by Perin et al. in a setting where verbal autopsies were the primary measure of cause-specific mortality. If this model were valid, it would have potential to estimate cause-specific mortality for high resolution age groups in countries where vital statistics are not complete or not of high quality.

## Methods

### Mortality surveillance

Data for this analysis come from the Nepal Oil Massage Study (NOMS). This was a randomized trial to assess the impact of two types of oil used to massage neonates on neonatal mortality in the rural southern plains of Nepal (trial is registered at Clinicaltrials.gov (NCT01177111)). Study data and methods are described elsewhere [9, 10]. A census of households in 34 Village Development Committees (a common geo-administrative unit in use at the time of the study) was undertaken to identify all married women of reproductive age. These women were visited every 5 weeks to determine if they had menstruated since the last visit. If they had not, they were offered a pregnancy test, and if identified as pregnant, were enrolled in the trial (as long as they consented). Women were followed monthly during pregnancy until delivery. As soon after delivery as possible, study staff visited the woman in her home to collect information about labor and delivery, and vital status of the neonate. The household was visited on days 3, 7, 10, 14, 21, and 28 after delivery to check on the infant and record vital status. If the baby had died, a verbal autopsy interview was conducted with the family after a suitable mourning period had lapsed. The verbal autopsy was adapted for use in this rural Nepali context from the 2007 WHO verbal autopsy instrument for neonates. InterVA-5, which uses a Bayesian mathematical model (developed to align with the 2016 WHO VA instrument as well as prior versions) was used to identify causes of death [11]. The trial was conducted from November 2010 through January 2017.

### Statistical methods

Neonatal deaths captured by mortality surveillance were recorded, including the time of birth and death (to determine the age in hours) as part of the verbal autopsy interview. Age of death of neonates was estimated as the difference between these date-times. Primary age groups were estimated based on the distribution of ages among deaths, with the objective of having approximately equal sized groups also carrying programmatic relevance (e.g. first day, first week).

The causes of death among neonates were estimated based on responses to verbal autopsy interviews with caregivers, which were processed by the InterVA5 algorithm. InterVA5 has the capability of assigning multiple causes of death for a specific death, although here we use only the underlying cause identified by InterVA5 for each death [9]. Deaths with multiple assigned causes were incorporated in all analysis by considering only the underlying cause, and ignoring secondary causes. After age groups were identified and causes of death specified, the cohort was divided into 25 mutually exclusive groups based on when over the study time period the deaths occurred, such that each group died within a contiguous three-month time period. For each three-month period (quarter), cause-specific mortality rates were estimated for primary causes for each age group of interest.

The log-quadratic model was developed to use age patterns in mortality rates to estimate the age distribution across the lifespan in cases where it was incomplete [6]. A different log quadratic model was developed to estimate age patterns within the under-five age group [5, 12], which was then adapted for cause-specific mortality for children under five years [7]. This log quadratic model as specified for age and cause-specific mortality was fit to these 25 data points (three month periods of time, or quarters) with some adaptations. The adapted log-quadratic model defined by Perin et al. was based on the relation

logxq0,c=ax,c+bx,clog5q0,c+cx,clog5q0,c2+vx,ck

for age *x* and cause *c*, where cause-specific under-five mortality (_5_q_0,c_) and it’s square were the covariates, and the shape parameter *k* was estimated for an individual life table given the cause-specific neonatal mortality rate. In this analysis, we used the all-cause neonatal mortality rate in place of the cause-specific under-five mortality rate, so that our log-quadratic mortality rate was based on

logxq0,c=ax,c+bx,clognmr+cx,clognmr2+vx,ck

and the shape parameter *k*, as in Perin et al., was estimated based on the cause-specific neonatal mortality rate. We examine two versions of the log-quadratic estimated mortality rates, one assuming shape parameter *k* is zero (for the typical set of age and cause specific mortality rates), and one where shape parameter k is estimated separately for each life table, corresponding to a three month period or quarter, based on the cause-specific neonatal mortality rate.

We had 25 different life tables to estimate this log-quadratic model. In order to test the validity of this model, we used cross-validation, holding out the deaths from each quarter in turn, estimating the parameters of the log-quadratic model, and the age and cause specific mortality of the hold out quarter. The absolute difference between the estimated and actual mortality rates for each age group was determined, and averaged across all 25 hold outs for the average cross-validation error. We compared this cross-validation error to the error of a standard approach, which assumes a constant daily mortality rate for each cause based on the total neonatal cause-specific mortality rate. The statistical software R version 4.3.1 was used for all analysis [13].

## Results

**Fig 1:**
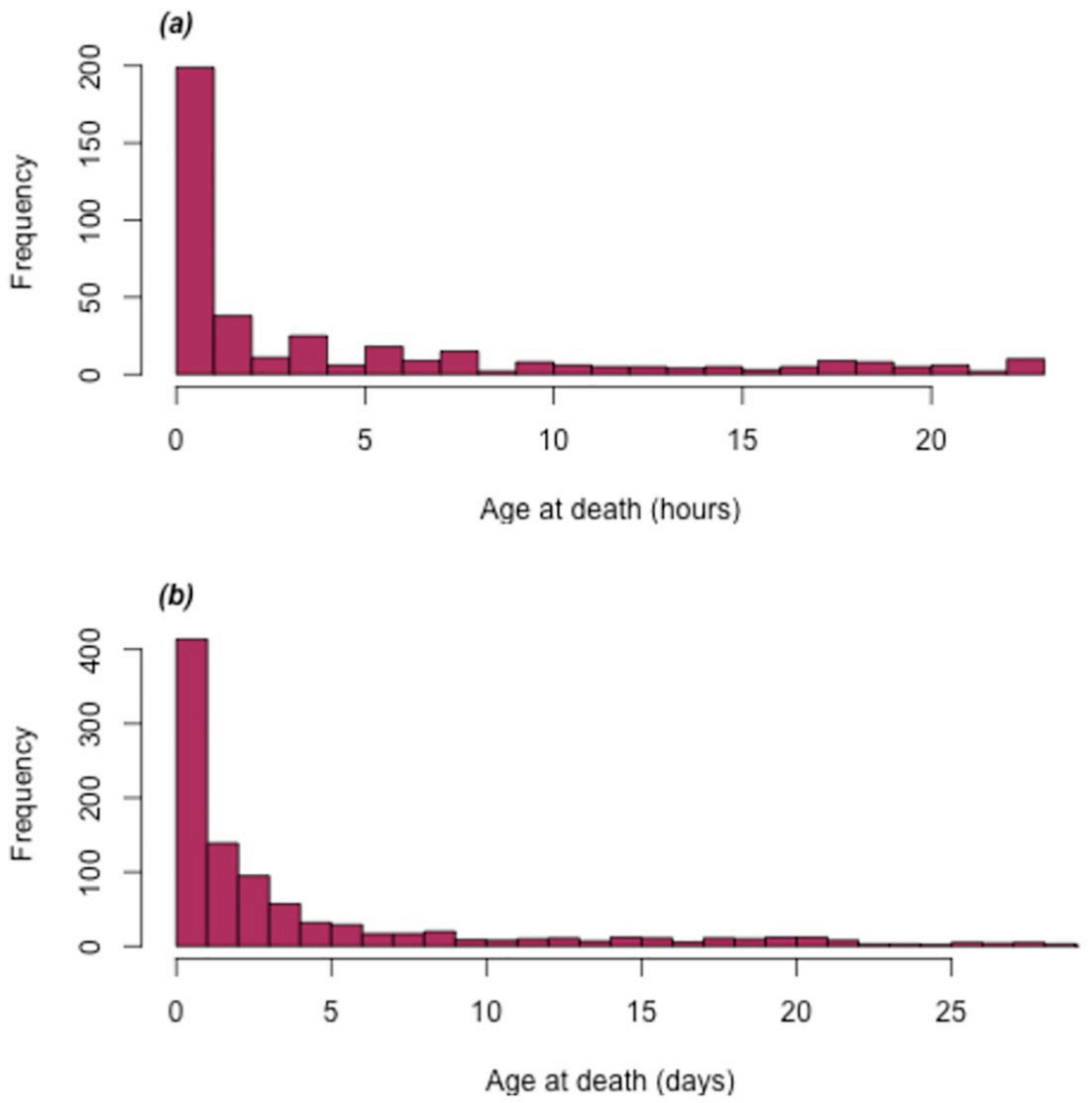
Age at death among (a) 397 neonatal deaths in the NOMS cohort who died within 24 hours after birth, in hours, and (b) 957 total neonatal deaths in the NOMS cohort, in days.

From the last quarter of 2010 until the end of 2016, there were 1007 neonatal deaths enrolled in the NOMS cohort, although 48 (5%) had unknown time of death, so age in hours could not be determined, and two neonates (0.2%) did not have verbal autopsies, leaving 957 (96%) for analysis. Over the study period, the all-cause neonatal mortality rate declined somewhat, from 35.3 deaths per 1000 livebirths in 2010 to 27.9 deaths per 1000 live births in 2016. We examined the age at death in hours for these 957 neonates, 397 (41%) of whom died during their first 24 hours of life. During the second day of life (24 up to 48 hours), there were 142 (15%) deaths. Thereafter, the daily mortality rate declined, such that 235 (25%) and 183 (19%) deaths occurred during 48 hours up to one week, and one week to four weeks of life, respectively. The complete distribution of approximate age at death is shown in Fig 1. We grouped neonatal mortality into four age groups for further analysis. We chose these age groups to have a similar number of deaths and also for programmatic relevance: less than 24 hours, 24 to 47 hours, 48–167 hours (“48h to 6 days”), and 168–672 hours (“1 week to 4 weeks”).

**Fig 2:**
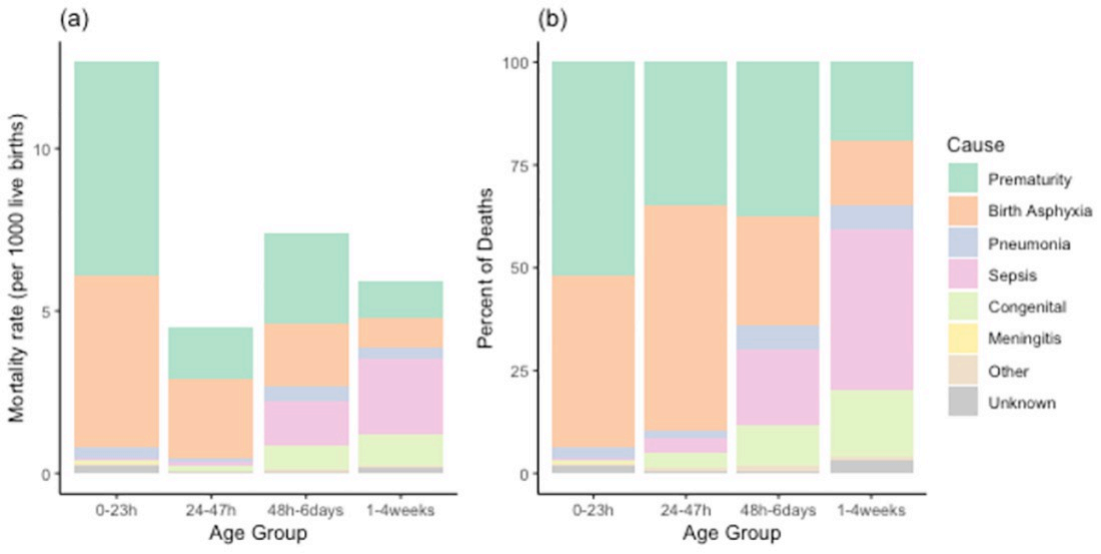
Cause of death by age as recorded for 957 neonatal deaths in the NOMS cohort, shown as (a) the number of deaths and as (b) the percent of deaths. Note that age groups are not equally spaced.

The predominant cause of death overall was premature birth (40%), followed by birth asphyxia (36%) and neonatal sepsis (13%). However, there was considerable variability of these causes across age (Fig 2). For those dying in the first day of life, prematurity was again the primary cause of death at 52%, followed by birth asphyxia at 42%. For those dying in the second day of life, birth asphyxia was the primary cause of deaths at 55%, while 35% of deaths in this age group were due to prematurity. For those dying in the latter first week of life (48 hours to one week), the most common cause of death was again prematurity at 38%, and birth asphyxia at 26%, closely followed by sepsis at 18%. For the older age group, those dying at one week up to four weeks of age, sepsis was the predominant cause of death at 39%, followed by birth asphyxia at 16%.

We estimated the log-quadratic model for the five most common causes of death (prematurity, birth asphyxia, sepsis, pneumonia, and congenital malformations). We expected the model to be the most challenging in the less common causes such as sepsis and pneumonia, because some age groups few deaths would be more common, and so fewer data points would contribute to the model for estimation.

We used hold one out cross validation to determine model performance, where a hold-out was one three-month period of time during the 25 quarters from late 2010 up to the end of 2016. We removed each of these 25 quarters in turn and estimated the log quadratic model for prematurity, birth asphyxia, sepsis, pneumonia, and congenital malformations with the remaining 24 quarters. We estimated the cross-validation error of these five models by taking the difference between the estimated and observed mortality rate for each hold out quarter. The resulting average and average relative cross-validation error is shown in Table 1.

**Table 1:** Absolute and relative cross-validation error for single hold-out time periods (25 unique 3-month periods or quarters), among 957 neonatal deaths in the NOMS cohort.

Age	Average Absolute Error			Average Relative Error		
	With k = 0	With best k	Standard*	With k = 0	With best k	Standard*
***Birth asphyxia***						
0-23h	0.00225	0.00225	0.00527	0.461	0.461	0.915
0-47h	0.00226	0.00203	0.00764	0.283	0.252	0.898
0h-6 days	0.00280	0.00111	0.00744	0.281	0.119	0.713
0h-4weeks	0.00276	0.00000	0.00000	0.263	0.000	0.000
***Preterm***						
0-23h	0.00239	0.00239	0.00585	0.470	0.470	0.914
0-47h	0.00276	0.00209	0.00708	0.525	0.279	0.878
0h-6 days	0.00259	0.00090	0.00781	0.291	0.098	0.713
0h-4weeks	0.00264	0.00000	0.00000	0.292	0.000	0.000
***Pneumonia*** **						
0-23h	0.00389	0.00389	0.00008	-	-	-
0-47h	0.00261	0.00020	0.00024	-	-	-
0h-6 days	0.00115	0.00077	0.00068	-	-	-
0h-4weeks	0.00166	0.00000	0.00000	-	-	-
***Congenital*** **						
0-23h	0.00047	0.00047	0.00043	-	-	-
0-47h	0.00060	0.00057	0.00054	-	-	-
0h-6 days	0.00108	0.00392	0.00074	-	-	-
0h-4weeks	0.00154	0.00000	0.00000	-	-	-
***Sepsis*** **						
0-23h	0.00004	0.00004	0.00015	-	-	-
0-47h	0.00018	0.00020	0.00029	-	-	-
0h-6 days	0.00119	0.00303	0.00086	-	-	-
0h-4weeks	0.00203	0.00000	0.00000	-	-	-

* Using average daily rate across whole neonatal period

** Small offset added to quarters with zero cumulative mortality due to sepsis, pneumonia, and congenital (1 x 10E-5), relative bias not included because observed mortality rate is often zero.

**Fig 3:**
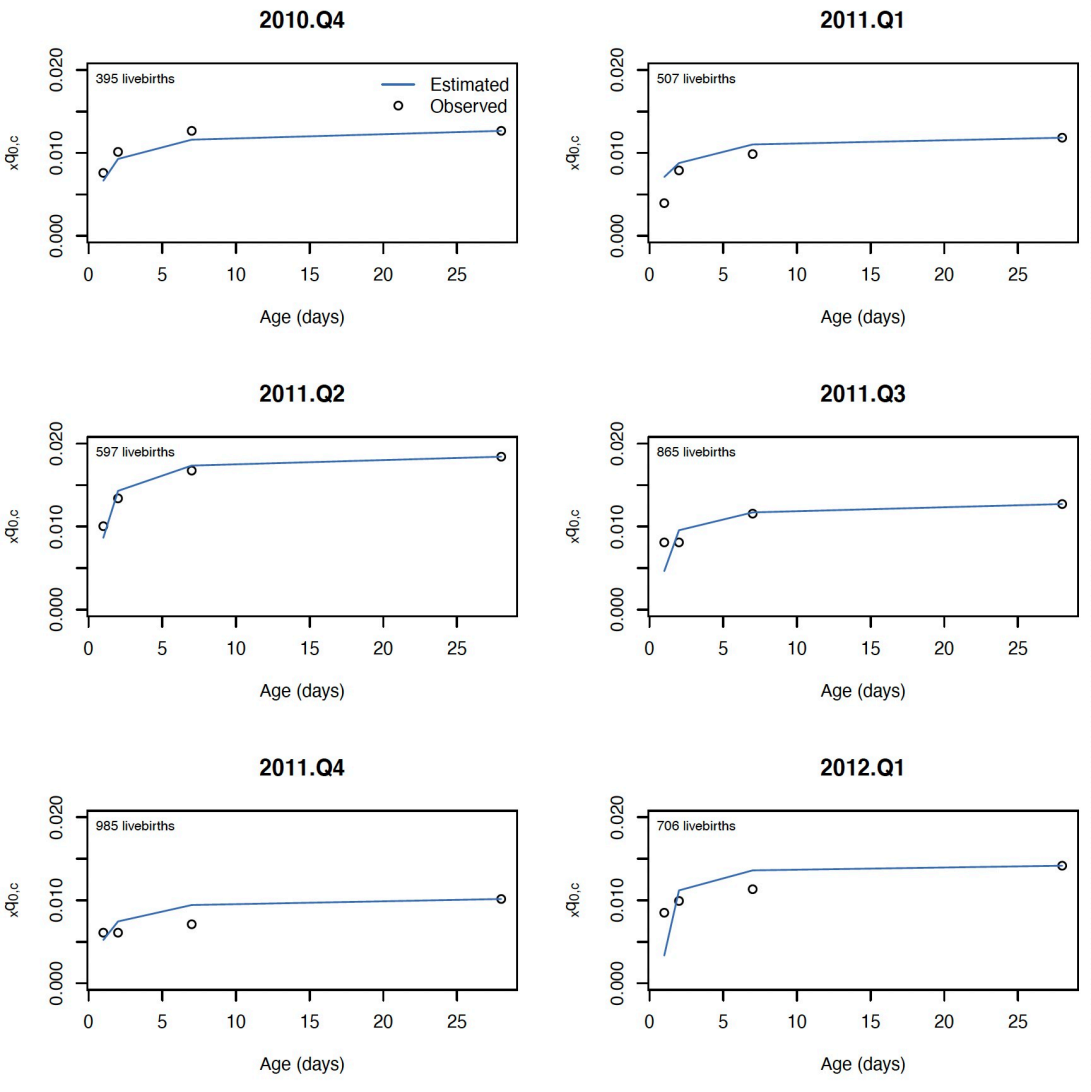
Estimated and observed mortality rate due to birth asphyxia for six consecutive quarters in the NOMS cohort.

**Fig 4:**
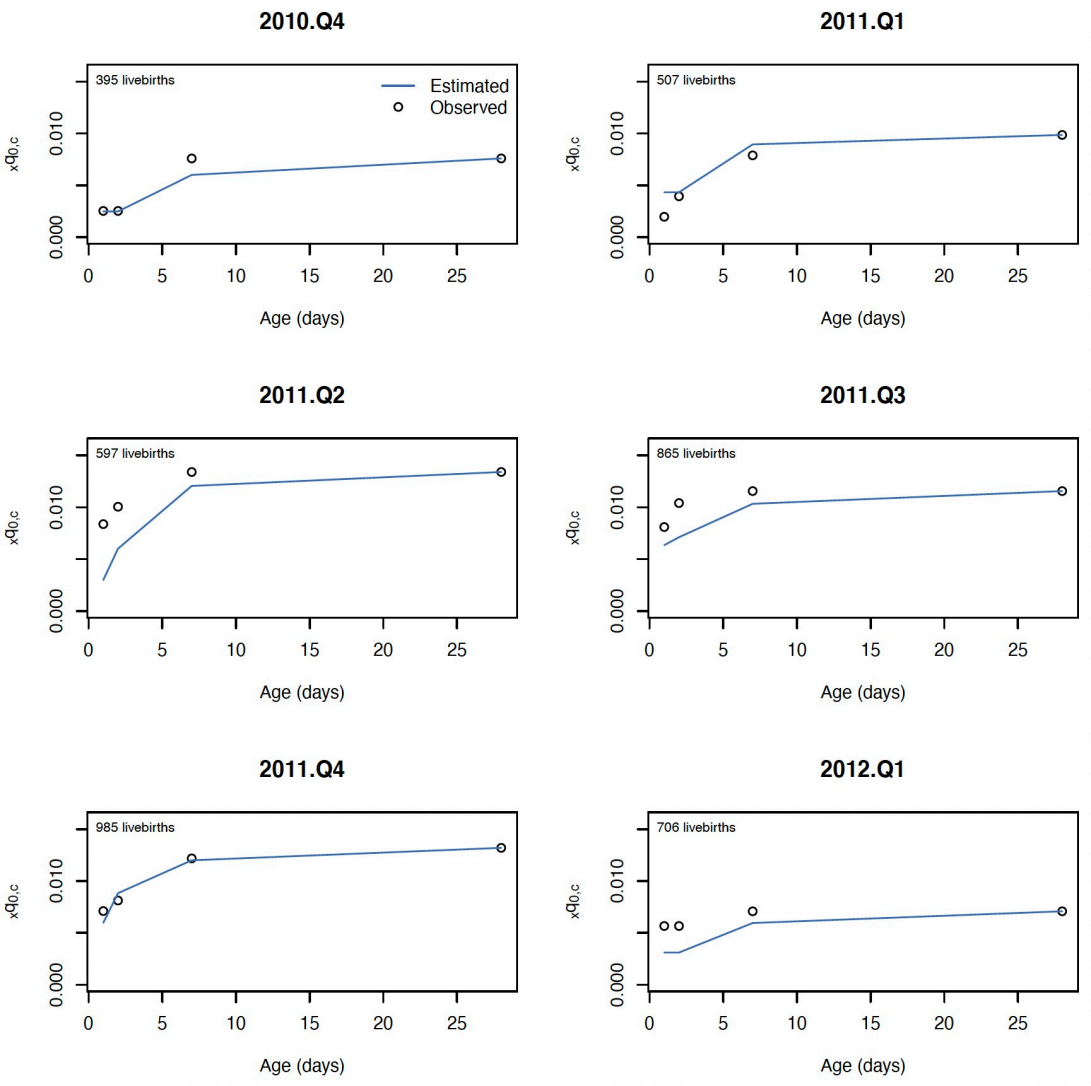
Estimated and observed mortality rate due to prematurity for six consecutive quarters in the NOMS cohort.

The log-quadratic model estimated the mortality rates in four age groups with similar error for birth asphyxia and prematurity, with average error ranging from 0.002 (2 deaths per thousand) to 0.003 for both causes when shape parameter *k* was assumed zero, and from 0.001 to 0.002 when shape parameter *k* was estimated for each hold out quarter. Generally, cause-specific mortality rates for younger neonates had larger cross-validation error, as expected, given the age groups considered were cumulative over age. Both versions of the log quadratic model performed favorably to the standard approach for estimating mortality rate for age-specific cause mortality, which had cross-validation error ranging from 0.005 up to 0.008. The cross-validation error relative to cause- and age-specific mortality rates was also similar for birth asphyxia and prematurity, ranging from 12% to 46% for birth asphyxia and 10% to 47% for prematurity. Estimated mortality rates for a selection of hold-out quarters are shown in Fig 3 for birth asphyxia and Fig 4 for prematurity.

**Fig 5:**
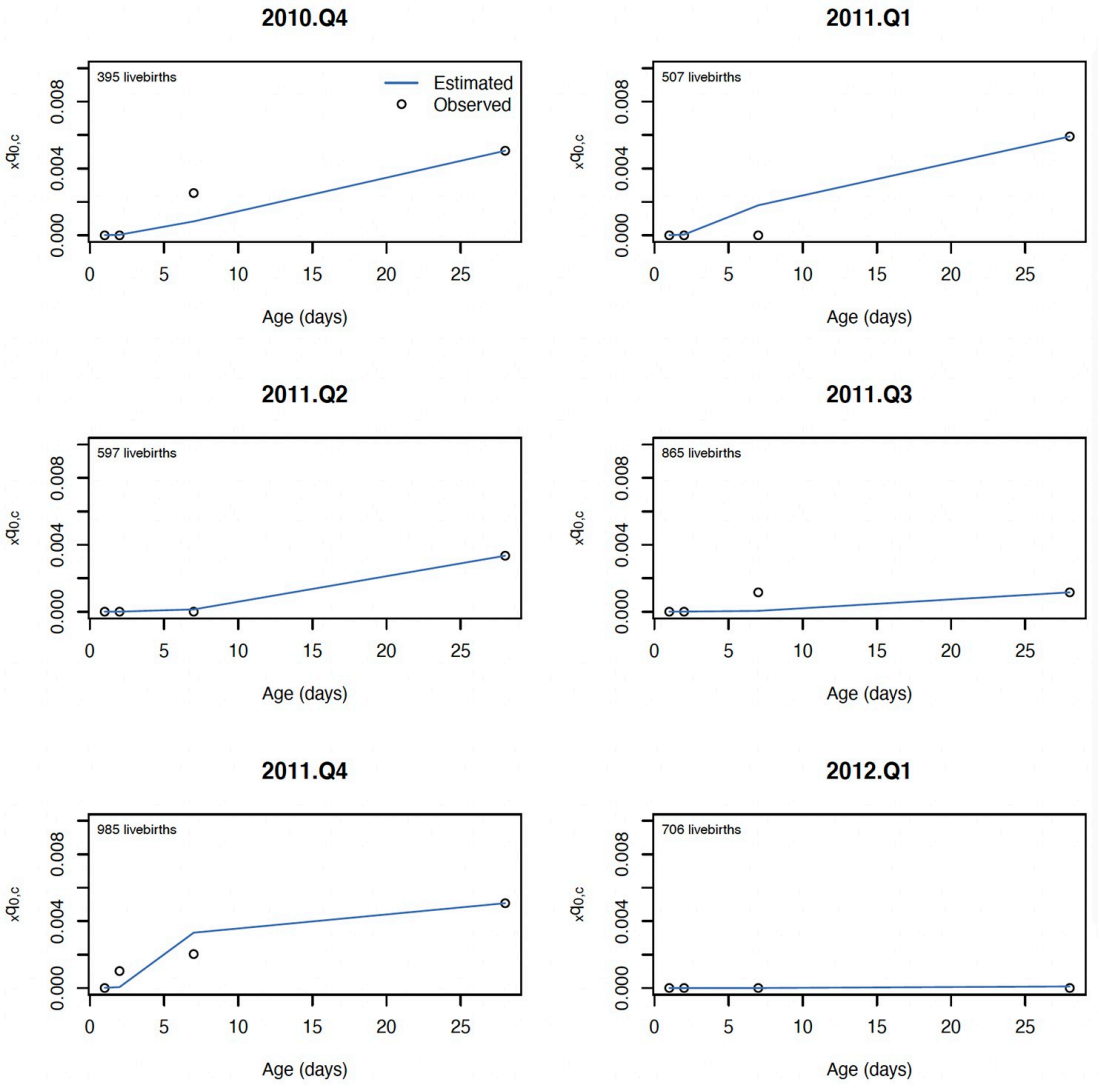
Estimated and observed mortality rate due to sepsis for six consecutive quarters in the NOMS cohort.

We also estimated the log quadratic model for sepsis (Table 1). The average cross-validation error for the log-quadratic model was lower for the first two age groups considered, however, for the third age group (when sepsis is more predominant as a cause of death), the log quadratic model had higher cross validation error on average, at 0.003 (three deaths per thousand), compared to 0.0009 (nine deaths per ten thousand) for the standard approach for age and cause specific mortality. Mortality rates estimated by the log quadratic model for a selection of quarters is shown in Fig 5. Cross validation error for pneumonia and congenital-specific mortality was slightly higher in the log-quadratic model across age groups compared to the standard approach. Mortality rates for congenital-specific mortality and pneumonia-specific mortality are shown in S1 and S2 Figs, respectively.

## Discussion

We adapted the Guillot et al. log quadratic model for estimating age and cause-specific mortality in neonates in a setting with high neonatal mortality, where the causes of death were ascertained by verbal autopsy. We summarized neonatal mortality in three calendar month time periods in four age groups as input to the adapted log quadratic model, basing the estimate of age and cause specific mortality on the log and the square of the log of the all cause neonatal mortality rate. The estimates of age and cause specific mortality rates were improved considerably relative to the standard approach for causes of mortality which were highly variable across age such as preterm birth and birth asphyxia, although this advantage was apparent for causes of mortality which were more evenly distributed across age such as pneumonia and congenital malformations.

Using the average cross validation error as a guide, the age and cause-specific mortality estimated here was not as accurate as that estimated by a similar adaptation of the log quadratic model for larger age groups using MCHSS data in China [7]. In China, pneumonia specific mortality for the first week was estimated within 0.65 deaths per 1000 live births, while here we estimated the pneumonia-specific mortality for first week of life within 1.15 deaths per 1000 live births. This is not unexpected, for several reasons. First, the surveillance system in China covered a larger population than the NOMS study, so there were more births and deaths contributing to the log quadratic model using the MCHSS data compared to that for the NOMS study, and thus mortality rates were more precise. The surveillance system in China, given its coverage, was divided into 120 strata years with over 65 thousand deaths for six age groups [8], in contrast to the NOMS study presented here, where we have 25 time periods with 957 neonatal deaths for estimating four age groups. The cause of death profile in China for neonates is also different from the NOMS study, with a lower fraction of neonatal mortality due to preterm and sepsis, and more deaths attributable to congenital malformations. In addition to these differences, since the MCHSS was based primarily on medically registered causes of death and the NOMS study used verbal autopsy for determining cause of death, there is likely more measurement error in the cause of death ascertainment for NOMS. For example, congenital anomalies were likely classified more systematically in the MCHSS, since verbal autopsies generally under identify congenital malformations [14].

The use of verbal autopsy in the NOMS study is both a limitation and a strength of this analysis. It represents first a limitation, because any assessment of error in the age and cause specific mortality is necessarily determined using the causes of mortality measured by InterVA5, which are not exactly aligned with the true causes. This is also a strength of our analysis and model assessment, given that most of the cause-specific mortality being measured in high mortality areas uses verbal autopsies, and so this is an important environment in which to understand the applicability of the adapted log quadratic model. The areas and countries where age and cause-specific estimates are most likely to be used by policy makers and health system managers have high mortality, where most cause-specific mortality is measured with verbal autopsies. Thus, measurements from verbal autopsy are a critical input in constructing a framework for estimating cause and age-specific mortality.

Our analysis has other limitations. Although we estimated the age and cause-specific mortality for five separate causes, these were modeled separately and so were not constrained by the all-cause mortality envelope. We expect that some precision would be gained by incorporating the all-cause mortality constraint in the process of estimating age and cause-specific mortality, however, the feasibility and potential benefit of this were outside the scope of this research. Rather, we aimed to understand whether an existing adaptation of the log quadratic model could be used to estimate cause specific mortality with a high resolution in age among neonates, and if this adaption could employ mortality surveillance information when the causes of mortality were estimated with verbal autopsy.

Our analysis also has strengths. We used mortality surveillance data from a large and robust surveillance system in a high mortality area of rural Nepal, which had both high quality follow-up of neonatal mortality and high resolution in the age at death for participating neonates. We used a systematic approach for identifying age groups and causes of death for analysis, with previously identified and validated methods for estimation, as well as robust methods with out of sample cross validation error for estimating the accuracy of our estimated mortality rates. Causes of neonatal mortality have been examined at high resolution in age previously in a diversity of settings [15, 16], including Nepal [17]. However, to our knowledge, this is the first study attempting more generally to model or estimate the cause-specific neonatal mortality with such high age resolution in low resource areas [18].

We have approximated the expected precision of high resolution age and cause-specific mortality estimates from the adapted log quadratic model. However, this may be an underestimate of the expected error of our model in practice, because the log-quadratic model requires a reliable set of reference data that can fairly represent the area for which cause-specific mortality estimates are developed. Here the high quality data we used for prediction was in a different time period, but from the same site. In order to use this model in areas having no primary data with high age or cause resolution, appropriate reference data would first need to be identified.

In theory, if reference mortality data could be identified, the adaptation of the log quadratic model presented here has the potential to estimate cause specific mortality for these age groups for other countries where vital statistics are incomplete. Sample surveillance systems such as COMSA, for example, measure age at high resolution and high quality cause of death for neonates [19]. Indirectly measured neonatal mortality and causes of neonatal mortality are also collected at high age resolution as part of some household surveys such as the Demographic and Health Survey (DHS), although age heaping may present challenges [20]. Further research is needed into the differences in age and cause specific mortality patterns and how currently available information from surveillance systems can be used to best inform health systems and decision making. The current standard for health system planning is to assume a constant cause distribution across the neonatal period [21], although this appears contrary to the evidence in many settings [22] including Nepal.

## Supporting information

S1 Fig Estimated and observed mortality rate due to pneumonia for six consecutive quarters in the NOMS cohort. (TIF)

S2 Fig Estimated and observed mortality rate due to congenital malformations for six consecutive quarters in the NOMS cohort. (TIF)
