# Supplementary figures and images for: Adapting the log quadratic model to estimate age- and cause-specific mortality among neonates
Source: PLoS One. 2024 Jul 12;19(7):e0304841. doi: 10.1371/journal.pone.0304841 (PMC11244816; doi:10.1371/journal.pone.0304841)

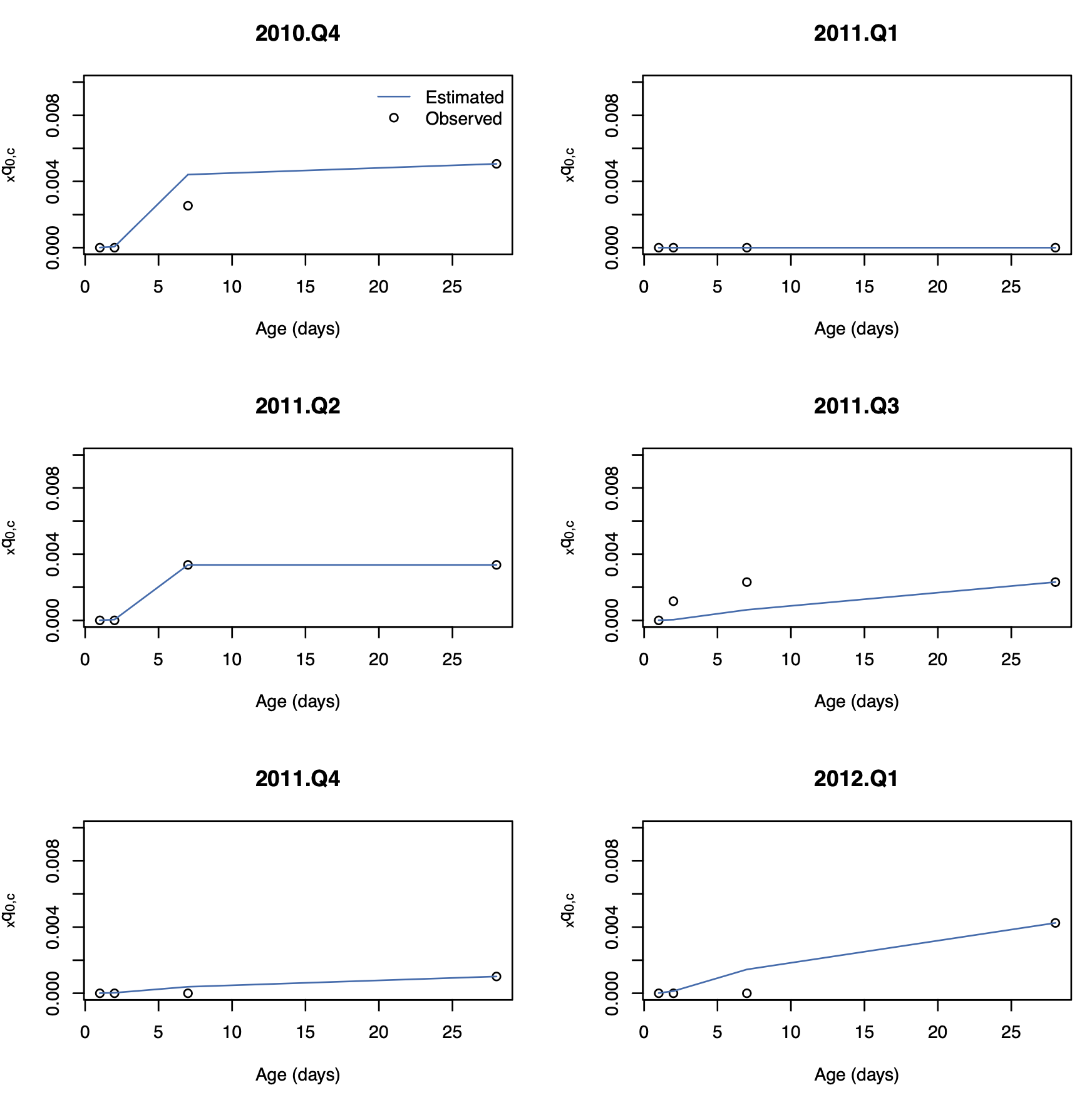

Supplement: S1 Fig — (TIF) [file pone.0304841.s001.tif]

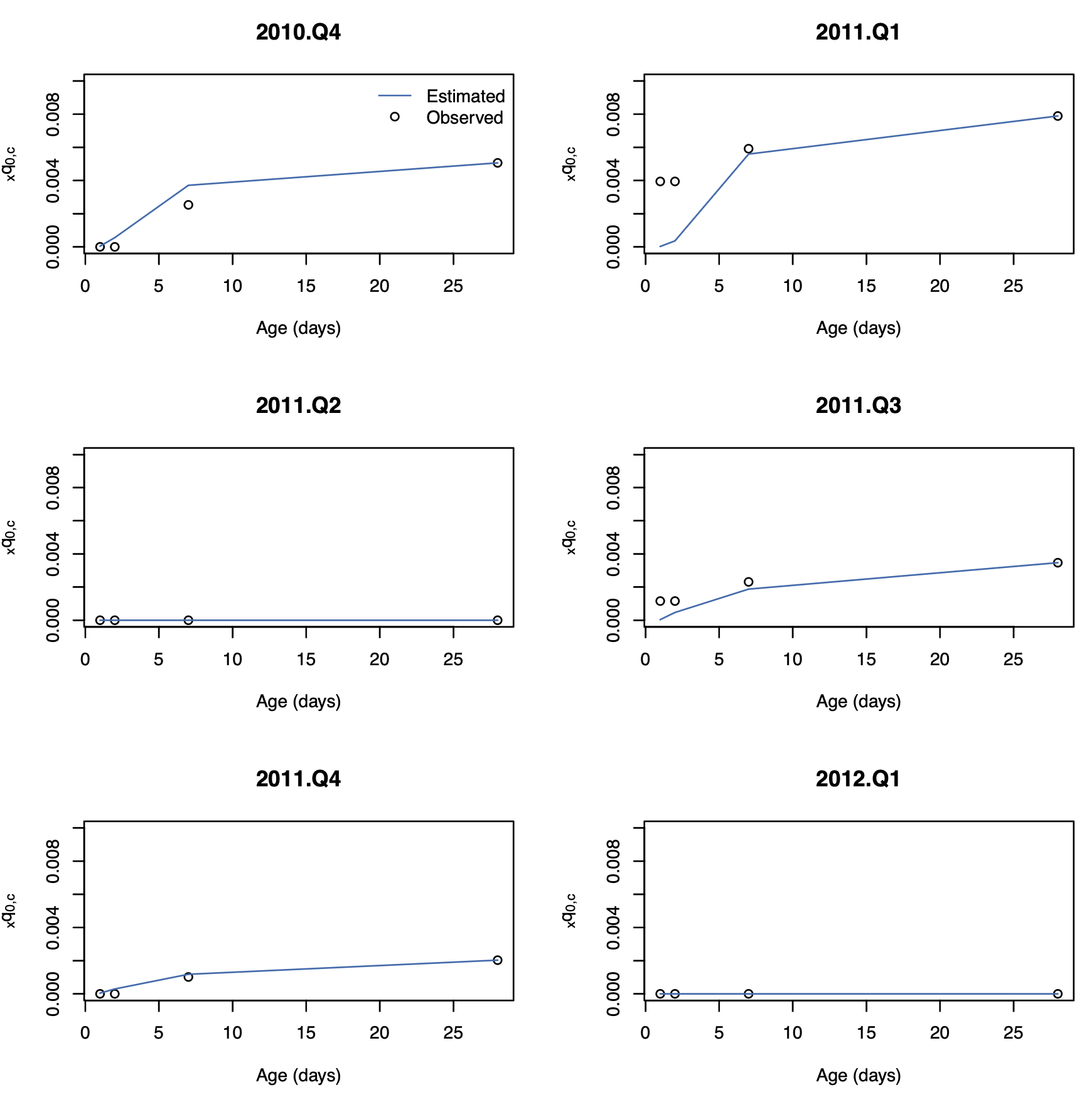

Supplement: S2 Fig — (TIF) [file pone.0304841.s002.tif]
